# Hope for the forgotten poor: Chinese male migrants, affective labor and the livestreaming industry

**DOI:** 10.3389/fsoc.2025.1640234

**Published:** 2025-09-01

**Authors:** Eileen Y. H. Tsang, Jeffrey S. Wilkinson

**Affiliations:** ^1^Department of Social and Behavioural Sciences, City University of Hong Kong, Kowloon, Hong Kong SAR, China; ^2^School of Journalism and Graphic Communication, Florida Agricultural and Mechanical University, Tallahassee, FL, United States

**Keywords:** affective labor, rural male migrants, guild, livestreamers, risks, China

## Abstract

In 2020, China's live-streaming industry reached an audience estimated to be 587 million users, which generated 961 billion yuan (Zixun, 2021). This technological transition to online commerce has enabled marginalized rural youth opportunities for career advancement and financial success as livestreamer hosts. This study examines how rural migrants capitalize on their affective labor to successfully engage their fans and supporters. Livestreaming guilds (*zhibo gonghui* 直播公會) play a pivotal role in recruiting, training, and managing online host personalities. Guilds monitor and manage the livestreamers by providing official reports of audience size, sales, and even how well the livestreamer performs. The career experiences of 62 rural-to-urban heterosexual male migrant livestreamers in China are examined. Affective labor is an immaterial form of labor that manipulates affect to generate feelings such as satisfaction, excitement, or passion in others. This article operationalizes affective labor to male heterosexual livestreamers using three distinct lenses: (1) sensuous dispositions tied to city life; (2) livestreamers' use of affective labor to maintain close relationships with their big tippers; (3) the relationships with the guilds and how livestreamers leverage affective labor to navigate their success and risks. This article offers a broadened perspective of rural-to-urban migrants in China. Through examining how young migrants become live-streamers, this article can provide insights into the evolving field of labor studies.

## Introduction

Yian (35), a former taxi driver, gracefully transitioned out of that labor-intensive industry in January 2017. The first author visited him at his Shanghai studio in January 2019. At 2 p.m., Yian began his livestream program through *Taobao* (淘寶, China's online shopping platform), talking to his audience and selling cosmetics like lipsticks, creams, and face masks. For 12 h, Yian interacted with the steady string of scrolling texts, alternating between questions about the products and compliments on his performance. He meticulously arranged the merchandise on a table, presenting them in a display case for optimal visibility. This arrangement facilitated a steady stream of instant purchases from his admirers. Once finished, Yian removed his headset, stepped away from the microphone and camera, and relaxed in front of the immense beauty lamp in his home studio. Before taking a sip from his ever-present water bottle, he picked up each of the three iPhones on his table to quickly respond and post his thoughts to more than 10,000 fans in the WeChat groups. Three assistants raced around him, cleaning up the room. Yian is widely known for his rural upbringing, unconventional behavior and free-spirit. His reputation is primarily anchored in the authenticity and credibility he has established through his streetwise experiences.

Yian is a successful livestreamer, the Chinese term trending within the nation's internet-based cultural and creative industries. Symbolically, the rags-to-riches stories offer hope for rural migrants from various occupations. Yian said that after the interview, he went to pick up a birthday gift for himself, a new Mercedes-Benz costing 2.8 million yuan (US$390,000). Yian's success has not gone unnoticed. In 2022, the population of migrant workers in China reached a staggering 295.6 million. Within this demographic, it is estimated that over 60 million individuals from rural regions engaged in livestreaming activities on e-commerce platforms, a trend closely monitored by the Ministry of Commerce (Shenzhen Statistics Bureau, 2019). Intrigued by Yian's success, a follow-up trip in December 2019 revealed that their stories are not uncommon. They sell legitimate products on social media platforms like Weibo (微博), WeChat (微信), Momo (陌陌), Bilibili (nicknamed B 站), Weidian (微店), Douyin, Kuaishou (快手), and Xiaohongshu (小红书). In 2020, there were at least 130 million livestream accounts in China, according to a report by the China Association of Performing Arts, and more than 600 million users of livestreaming-related services (BBC Trending, 2017; Li, 2022).

Some gaps in the socio-cultural Sociology literature are addressed through the lens of affective labor. First, the existing literature on livestreamers often focuses on gay male livestreamers (Wang, 2020a,b) and female livestreamers (Banet-Weiser, 2012; Liao, 2016; Senft, 2008; Wang, 2021; Yip, 2017). Second, some studies find that livestreamers are routinely exploited (Wang, 2021), with only cursory attention given to the benefits and consequences of being a livestreamer in the Chinese context. Qualitative research methods can best contrast the benefits with the consequences of being a livestreamer. For example, heightened self-esteem, being creative, and enjoying financial success must be balanced with the reality of long hours, high stress, uncertainty, and feeling manipulated or exploited. Third, there is limited research applying the concept of affective labor detailing how livestreaming guilds operate and manage male livestreamers.

The concept of affective labor (Hardt and Negri, 2004) zooms in on the effect experienced between the livestreamers and audiences and how they navigate their emotions and expression—wellbeing, satisfaction, and excitement—in livestreaming. By examining how individuals navigate their emotions and expressions, the study sheds light on the intricate dynamics of impression management within this specific social context. The emergence of livestreamers as a new profession, borne out of technological advancements and globalization, opens fresh avenues for understanding shifts in gender roles, labor dynamics, and sexuality in China. The livestreaming industry is controlled and managed by guilds. The guilds sign and support livestreamer hosts and manage them closely, including audience growth, churn, and decline (Liu et al., 2021). The affective experiences of livestreamers in China reveal overlooked industry dynamics and underscore the intricate interplay of gender, class, and power. Despite the significant role of guilds, the Chinese government has not established comprehensive professional ethics for their governance. As a result, livestreamers must rely on themselves rather than hoping for government intervention. This reliance highlights the need for individual agency and collective support within the community, allowing streamers to navigate complex socio-economic challenges independently for urban migration. These insights emphasize the importance of understanding affective labor within digital economies amidst insufficient institutional support.

The primary research questions are: (1) What are the most conspicuous forms of affective labor used by male livestreamers to contribute to their success and exploitation that differ from female and gay livestreamers? (2) How do male migrants capitalize on affective labor to become livestreamers to maintain their success with the big tippers? (3) How do the guilds manipulate the male livestreamers and affect their success and exploitation?

This article examines the burgeoning phenomenon of heterosexual male livestreamers in Shenzhen, focusing particularly on their use of affective labor to excel in the livestreaming industry and ascend the social hierarchy. Affective labor, which involves the management and production of emotional experiences, is instrumental in the monetization strategies these livestreamers employ. Utilizing ethnographic methods, including thick description, interviews, and participant observation, this study gathers qualitative data from these male livestreamers to understand how they navigate the highly competitive industry. Through participant observation, interviews, and content analysis, the research delves into the daily routines, challenges, and strategies these individuals use to sustain and expand their online presence.

The affective dimension not only influences the personal lives of livestreamers but also provides a lens through which the intersections of gender, class, and power are prominently displayed. These intersections affect the dynamics within streaming guilds and inform the self-help initiatives undertaken by the streamers. It is suggested that the livestreaming industry should promote self-help programs rather than relying solely on the Chinese government for protection and regulation. Although guilds play a significant role in regulating the industry, the Chinese government has not yet established a comprehensive set of professional ethics for guild governance. Further details can be found in the conceptual framework and methods section. Focusing analysis on the intersectionality of affective labor and gender provides a robust theoretical framework for understanding broader societal dynamics. It compels us to question the intrinsic economic and cultural valuations of labor and to recognize how these valuations are shaped by gendered associations. By scrutinizing how affective labor is valued based on its association with femininity, scholars can challenge entrenched economic paradigms and contribute to a reimagining of labor value systems. Such an intervention not only enriches gender theory but also carries the potential to inform policy and practice, advocating for a more equitable recognition and remuneration of affective labor across all gender identities.

The organization of this article first presents the concept of affective labor and how male migrants capitalize on emotion and affection in their careers to navigate their success and exploitation. Next, a description of the research methods used to collect data during two field site excursions, followed by the analysis of interview data. Lastly, we conclude by analyzing hegemonic masculinity and manipulation by the guild and suggest self-help programs in China.

## Conceptual framework-affective labor

The affective labor concept originated from Hochschild (1983) and Goffman (1959) in Western countries. Hochschild (1983) asserts that emotional labor necessitates the coordination of mind and feeling, often drawing from a deep, integral source of self. In performing affective labor, livestreamers present an appealing persona to foster a sense of community through real-time viewer interactions. This labor requires careful emotional management, mirroring Hochschild's (1983) concept of surface acting, where outward emotional expressions are adjusted without altering internal feelings. Livestreamers manufacture positive emotions to attract and engage viewers. Success often depends on forming emotional connections with viewers, involving deep acting, where individuals strive to align their internal feelings with the expected emotional displays.

Moreover, affective labor in livestreaming resonates with Goffman (1959) dramaturgical theory of social interaction, which likens social life to a theatrical performance. Livestreamers' affective labor involves managing emotions to construct an appealing persona, reflecting Goffman's “front stage” behavior, where individuals perform specific roles in public regardless of their actual feelings. When they are off-camera and not streaming, livestreamers may experience different emotions, reflecting Goffman's “backstage” behavior, where individuals can be themselves away from public scrutiny. Livestreamers must manage their emotions and perform in ways that meet audience expectations while also dealing with their own personal feelings and experiences (Hacking, 2007; Li, 2019).

Previous research on affective labor on livestreamers is comparatively more focused on female and gay livestreamers than heterosexual male livestreamers. The literature on affective labor often examines professions perceived as feminine (Banet-Weiser, 2012; Senft, 2008; Ye et al., 2022). In China, research on affective labor has been heavily gendered, focusing on female livestreamers catering to the emotional needs of “unattractive, lonely leftover men” (Cunningham et al., 2019; Tan and Shi, 2021). Studies on gay livestreamers have confronted structural barriers related to their non-toxic masculinity presentation, appearance, and performance (Wang, 2020a). Both groups have successfully attracted big tippers through sex appeal alone (Wang, 2020b).

However, this article seeks to extend the literature by noting how heterosexual male migrants can also leverage affective labor to achieve success in the livestreaming industry (Tan et al., 2020). In addition, the literature has identified specific practices which exploit livestreamers, but rarely have studies examined the activities that help them to be profitable. This article argues that rural migrants go into livestreaming as entrepreneurs, consciously working with the guilds and earning tips from followers and fans. Livestreamers in the West are known to present their “authentic selves” across various platforms to attract a wider audience (Nayar, 2017; Senft, 2008; Usher, 2020). This emotional investment helps create and maintain a genuine relationship with viewers, with elements of both success and exploitation (Cunningham et al., 2019; Tan and Shi, 2021; Xia, 2021; Zhang et al., 2019). Lastly, the literature seldom mentions details about the critical role played by the livestreaming guild. The relationship between the livestreamer and the guild is complicated, interactive, and reciprocal. Guilds collaborate with algorithm experts to endorse products and brands that will be advertised on platforms (Bishop, 2020). Livestreaming guilds control entry into the sector, providing needed support such as training, meals, accommodation, and marketing (Zhang et al., 2019). They also set targets for sales and audience size, and provide regular reports on audience analytics, sometimes even manipulating platform algorithms and audience preferences (Liu et al., 2021). Meanwhile, guilds monitor livestreamers for text-based censorship, filtering hashtags for sensitive keywords (Knockel et al., 2015). The reciprocal efforts of the guild trying to control versus the livestreamer's efforts to succeed reflect the dynamic concept of affective labor.

The frameworks developed by Hochschild (1983) and Goffman (1959) help us understand the importance of affective labor among male migrants in the context of livestreaming. Affective labor involves the creation and manipulation of social emotions and atmospheres, transcending the boundaries of traditional emotional labor, which is typically confined to managing personal emotions within service roles. Unlike immaterial labor, which broadly encompasses the production of non-tangible goods like knowledge and information, affective labor is specifically concerned with crafting the emotional and affective dimensions that influence social interactions and communal experiences (Hardt and Negri, 2004; Hardt, 1999; Liao, 2016; Yip, 2017). Affective labor includes non-cognitive responses to stimuli that create a sensory attachment to the livestreamers' work or geographic location (Dragojlovic, 2014). Livestreaming is highly competitive, exploiting the parasocial aspect of livestreaming to garner support from followers. Parasocial interactions through streamed performances build emotional connections and value, demonstrating “affective power” (Marshall, 2010). This emotional bond attracts and retains viewers, facilitating the rapid accumulation of substantial followings (Tan and Xu, 2020). To establish a working definition grounded in livestreamers in China, affective labor is exemplified through hosts who curate emotionally engaging narratives and cultivate a sense of belonging among their audiences, thus transforming spectatorship into participatory communities. This capacity to generate and sustain affective atmospheres is integral to viewer engagement and economic strategies in live-streaming, distinguishing it from mere entertainment or information dissemination.

This study focuses on three important contextual aspects; (1) sensuous dispositions tied to geographical space reflecting how the spatial environment reinforces an impression of cosmopolitan urban success; (2) livestreamers' use of affective labor to maintain close relationships with their audiences, especially those who are considered “big tippers”; (3) the relationships with guilds and how livestreamers leverage affective labor to navigate their success and setbacks.

Public performance invariably involves an element of risk. Livestreamers must pay high commissions to guilds at exorbitant interest rates, navigate political surveillance, and rapidly assess their performance and audience connection. The livestreaming environment is inherently uncertain, necessitating self-management practices associated with various forms of physical, emotional, and embodied labor (Kleppe, 2017). Guilds initially set all the rules and conditions for livestreamers regarding pay, working conditions, product lines, and services sold.

## Methods

This study employed a qualitative approach because of the thick description needed to address the research questions (Geertz). Thick description enriches the narrative and interpretive depth of the study, allowing for a more nuanced understanding of the contextual layers and meanings that participants ascribe to their experiences (Bernard, 2011; Riessman, 1993). Clifford Geertz's concept of “thick description,” as introduced in Geertz (1973), is a pivotal methodological approach in qualitative research, particularly in anthropology. This concept emphasizes the importance of detail and context in understanding cultural phenomena. Thick description requires researchers to go beyond surface-level observations by immersing themselves in the social and cultural contexts of their subjects. This approach involves capturing not just the actions and behaviors of individuals but also the meanings and interpretations that they assign to these actions within their specific cultural settings. In qualitative research and interviews, applying thick description means engaging deeply with participants, encouraging them to share comprehensive narratives that reveal their underlying motivations and perspectives. Researchers must be adept at interpreting both verbal and non-verbal cues, situating them within a broader socio-cultural framework. Through this method, the research provides a holistic and nuanced account, revealing complex interplays of meaning and offering rich insights into the lived experiences of participants.

Data was collected in two excursions, interviewing male migrants in Shenzhen who transitioned into livestreaming careers. The first data collection period (January 2014 to December 2017) involved interviews with 201 migrants in unskilled jobs, many of whom expressed an aspiration to become livestreamers. The second data collection period (January 2018 to December 2019) involved follow-up interviews with those initial 201 participants, 62 of whom had successfully transitioned into livestreaming. The livestreamers ranged in age from 18 to 35 and had various unskilled labor work experience. Before entering livestreaming, 19 had worked in factories, 8 had been masseurs, 11 had sales experience, 2 had been fashion models, and 17 drove taxis. All 62 admitted they exclusively relied on the guilds in Shenzhen for training and management since they did not have social capital, skills, or reputation to become a livestreamer. The guild interviews potential livestreamers before offering them a contract. This contract delineates specific targets and monthly quotas. However, certain concealed aspects, such as the interest rate they owe to the guild and the required number of hits per month, remain ambiguous. Despite their transition into the realm of livestreaming, a significant number of participants continued to grapple with financial challenges. Roughly half of them reported moderate success, garnering an income exceeding 20,000 yuan (US$2,570) per month, a figure considered to represent a high salary. Conversely, the remaining participants reported an average monthly income of ~4,000 yuan (US$588). A monthly income below 2,000 yuan (US$307) is deemed a low salary. This disparity underscores the varying degrees of financial success within the livestreaming industry. All participants identified as heterosexual and originated from rural areas. Two-thirds had only a primary school education.

The guilds facilitated access to a diverse range of interviewees beyond the initial male migrant population. During the COVID-19 outbreak (January 2020 to January 2021), the first author corresponded with the participants via WeChat/Douyin to monitor career progress. Data collection methods included in-depth interviews, participant observation, casual conversations, online and offline activities, and life histories—data comprised of recorded interviews lasting approximately an hour, *in situ* note-taking, and post-event field notes. Interview questions included how they cultivated rapport with the audience and their most generous patrons, what motivated them to initially work as a livestreamer, and how best to characterize the relationship with the guild. Consent was obtained prior to any recorded interview, and participants could withdraw at any stage. Confidentiality and anonymity were assured, with pseudonyms used in place of real names, mobile phone numbers, and WeChat accounts.

The data was analyzed using grounded theory (Glaser and Strauss, 2017), an inductive approach that generates theory from qualitative data. Transcripts were translated into English and analyzed using NVivo 12.0. Preliminary coding involved repeated reading of transcripts to Initially, the coding process involved identifying significant phrases and expressions that directly pertained to the research questions or highlighted noteworthy experiences. This stage was crucial in distilling the raw data into a set of initial codes. Subsequent analysis led to the development of several categories or themes, such as heterosexual male livestreamers, gay livestreamers, female livestreamers, guild, risk, struggles, and algorithm. These emergent themes underscore the cultural specificity and nuanced dynamics within the livestreaming industry, particularly concerning how heterosexual male livestreamers navigate and survive in this environment. To ensure the robustness and validity of the identified patterns, several strategies were employed. These included iterative coding and constant comparison, enabling the refinement and validation of themes as the analysis progressed (Riessman, 1993). Furthermore, to enhance the reliability of the findings, peer debriefing and member checking were conducted, allowing for the verification of the themes by those who contributed to the dataset. This triangulation helped ensure that the themes accurately captured the participants' perspectives.

We identify themes such as the role of affective labor in risk-taking and interacting with the guild.

In conducting research involving sensitive topics, robust ethical and legal considerations are paramount. Talking to livestreamers proved to be very sensitive in a number of areas. Informed consent was obtained, ensuring participants were fully aware of the study's purpose, the voluntary nature of their involvement, and their right to withdraw at any time. To maintain confidentiality, pseudonyms were used, and identifiable information was meticulously removed from all data records. During interviews, a well-trained social worker from Hong Kong provided emotional counseling if livestreamers felt vulnerable, stigmatized, and marginalized (Tsang, 2020a; Tsang and Fang, 2024; Tsang, 2020b). Data was securely stored, with encryption and restricted access protocols in place. The study was also vetted and approved by an institutional ethics review board, aligning with established ethical guidelines to safeguard participants' wellbeing and the integrity of the research process.

### The city gives hope: sensuous attachment to the urban environment

Affective sensuous attachment to the city helped inspire many of these individuals to leave low-paying jobs to work as livestreamers. Although the 62 informants lacked a city *hukou*, they were eligible to live and work in the city. They all acknowledged that city life gave them opportunities that were unthinkable in their rural hometown villages. Fangyi (24) left his factory job in 2016 but stayed in Shenzhen studying yoga. He found Shenzhen to be dynamic and full of positive energy. Fangyi (24) successfully auditioned and began to livestream his yoga knowledge and massage skills. After 4 years, he enjoys a following of roughly 800,000, which he attributes to working with “heart, emotion, and affection.” He teaches yoga techniques to relax and reduce stress. He admitted that livestreaming is “passion and excitement, like getting high and riding a rollercoaster.” He says,

I am passionate about yoga. Shenzhen is such an incredible, global city full of promise. I love wandering around downtown areas like Guomao Road (國貿路). It fills me with hope and motivation. I often livestream myself doing yoga on the rooftop, surrounded by all the city noise and chaos. Shenzhen feels like a beacon of hope, smells like success, and sounds like the hustle and bustle of city life. And when I look around, I see the whole cityscape stretching out before me. It feels good…

Fangyi earned 12,000 yuan [US$1,846] per month, typical of many who use live streaming to promote a business. He brands himself by posting selfies on social media and livestreaming on Taobao and Douyou. For Fangyi, sensuous dispositions reflect the security and comfort he derives from the city's supportive environment. The city's sights, smells, and sounds convey to him a sense of opportunity that was missing in his rural village. The soul-searching technique works body muscles and builds self-confidence and self-appreciation.

Zhilan (29) began livestreaming his high-energy choreographed dance routines from Shenzhen in 2017 and was told by the guild he had 5 million followers by 2020. He had been bored with his low-wage masseuse job and felt it conflicted with the excitement of city life; even the architecture seemed modern and full of hope. The parks, shopping malls, cafés, and open atmosphere mirrored his desire to channel his own energy into something uniquely his. Zhilan says his livestream dancing mirrors the energy he finds in Shenzhen. He says,

I feel the hope of life and my sense of belonging; I can touch the vivid and vibrant city life; I can smell the fragrance of life and delicious street food; I can see the skyscrapers; I can hear the people singing and dancing in the commercial hubs. I livestream from my penthouse and enjoy the panoramic view… I immerse myself in the cacophony of the streets below. The distant echoes of life blend seamlessly with the urban symphony, much like the soothing whispers of waves along the vast Atlantic Ocean…I am swaying to the rhythm of the sky. My body, an instrument, resonates with the pulse of the concrete jungle, and for those ephemeral moments, I am both observer and participant in this bustling urban ballet…

From the conversations, affective labor and a sensuous disposition propel livestreamers toward achieving their life goals. The above conversation showed that affective labor manifests the profound hope and ambition for a better life—stirred by the promise of Shenzhen—a city emblematic of their aspirations. This emotional construct motivates them to navigate the challenges of livestreaming. The city's potential opportunities infuse them with a sense of excitement, further fueling their ambition. For these rural men, emotions play a crucial role in motivating their goals and helping them stay strong when facing difficulties (Tsang, 2020c). They believe that feelings like love and passion can lead to success. Feeling a sense of belonging encourages them to use their abilities to connect with others and enjoy life, which are key aspects of being human. By channeling their enthusiasm and excitement for their jobs, they can create value and achieve success (Coffey et al., 2018). In this context, the creation of sensations, emotions (Hochschild, 1983), or embodied experiences constitutes the actual product of their work (Hardt and Negri, 2004; Lazzarato, 1996; Marshall, 2010).

### Male livestreamer—follower relationship

Male livestreamers engage in affective labor to build and maintain positive relationships with their viewers, whether female, gay, or so-called big tippers. Many of the 62 interviewed said most of the viewers in their chatrooms are older single women, whom the livestreamers referred to as golden leftover ladies (剩女a woman who is over 27 but still single), and “rotten ladies” 腐女 (Japanese term for female fans of manga, anime, and novels that feature romantic relationships which cannot be achieved in reality and only fantasized about with livestreamers or film stars). Ziyi (28) transitioned from a fashion model to a livestreamer in December 2018. He followed the path forged by Li Jiaqi (a popular male livestreamer) selling lipstick from Taobao (Global Times, 2018). He gained notoriety by applying lipstick directly to his lips instead of the normal practice of applying it to the arm. But breaking that rule gave his performance indisputable authenticity (Tsang, 2021). He credited plastic surgery and naturally delicate skin with helping him successfully apply principles of consumer psychology (Elfving-Hwang, 2020). To keep their followers and big tippers (big brother, *dage* 大哥 and big sister, *dajie* 大姐) in the chatrooms, the livestreamers have to fulfill audience requests, like sending product samples and small gifts of appreciation. The livestreamers do not know when the request is genuine, a test by the manufacturer, or even the guild. The livestreamers maintain a nuanced gendered relationship with the *dajie* (Tsang, 2021; Tsang and Wilkinson, 2022). They must implicitly perform sexualized intimacy to keep their clients returning (Tsang and Wilkinson, 2022). Ziyi says,

I always try to present myself as friendly, charming, and approachable. No seduction (套路 *tuluo*), only genuine feeling. I even respond to comments and chat with them. When I ship products to my audiences, I always include a small card with a blessing or fortune and sometimes small gifts like samples of skin lotion. I have found that flirting is a great tool for keeping my audience engaged and entertained. This could involve complimenting viewers, making playful jokes, or using suggestive language. It is all in good fun. It keeps things lively, and my viewers seem to enjoy it. It is all about creating a bond with my fans and making sure they come back for more.

Guanyao (28), was a salesclerk in a local clothing boutique when he switched to livestreaming in 2016. He sold clothes in his livestreaming and maintained his figure to model the items he sold. He described a big tipper called Fanfan who used to always watched his livestream show. Once, after a show, she wanted him to dance and show his muscles. Guanyao said afterward they developed a friendly relationship, talking often past midnight. He treated her like a princess (*gongzhu* 公主) or queen (*nuwan* 女皇), and in the chat she called him dear husband (*laogong* 老公), while he called her sweetheart (*tianxin* 甜心). Guanyao says,

Competing with female livestreamers is tough. Women can use their looks and charm to win over the audience. Women are much better at flirting with male clients. If I lose a game, I have to strip down to my underwear, pretend to dance a striptease and act like a clown on camera to let the audience laugh and tease. It is nerve-wracking. I always worry my *dajie* and *dage* will leave and stop giving me tips and gifts. For this job, I must develop a sense of love and affection…luckily, I can get a tip from those female *big tippers* for $100,000 yuan (US$15,300). Therefore, I still need to use my heart to work for this job.

Guanyao reiterated many times during the interview that he avoided any romantic or sexual entanglements with his clients in the private chatroom. But the pressure for tips and gifts is ever-present, and frank requests for striptease and online sex for cash are routine. Guanyao characterized the older single ladies as more aggressive and sexually hungry than soldiers (*nuge bi shibing hai jiefang* 女客比士兵還解放). Women around 30 are like a tiger (*nuren sanshi ruhu* 女人三十如虎), and women over 40 are like a wolf (*nuren sishi rulang* 女人四十如狼). Therefore, Guanyao has to keep things from going too far while still flirting and playfully acknowledging the requests of his female clients who are older, confident, and rich. They stay online past midnight and provide tips electronically through Alipay and WeChat. Guanyao's narrative reflects his affective labor as a livestreamer. He works hard to please the women who demand his attention, hoping they will keep returning and keep his business profitable.

Besides the female patrons and clients, many livestreamers interviewed said there are a significant number of gay clients as well who refer to the livestreamer host as a “fresh flesh man” (*xiaoxianrou* 小鲜肉), which means young, cute, cheerful, and physically attractive. Gay clients also use the term “sunshine boy” (陽光男) for someone desirable who is upbeat and energetic. Since the livestreamers are focused on demonstrating and selling products or services, the shows are high-energy but sexually muted. Zhengyou (25) was formerly a factory worker in Shenzhen who now runs a dance studio in Guangzhou. He joined the guild in 2016 and by 2020 had around 200,000 followers. His nightly livestream (after 9:00 p.m.) from his brick-and-mortar dance studio routinely attracts big tippers because of his energy and versatility.

I pretend I dance like “The Renegade with ‘Lottery (Renegade),' WAP dance, ‘Cannibal,' and ‘Vibe (If I back it up).”' I must also perform Chinese opera and keep my hairnet, outfits, and manicured nails ready. I can grab my foundation and wig in seconds and transform into Chinese opera characters. Whatever the audience wants me to be, I do it in seconds.

Instead of being a simple farm boy from China's subtropical south, Zhengyou refers to himself as a successful “cosmopolite with a smartphone, skincare products, and fashionable clothing.” He joined the guild for the exposure to wealthy tippers. Sometimes the audience demands the livestreamers give them something extra. The guilds regularly organize PK competitions. The term “Player Kill” (PK), borrowed from gaming, refers to a competitive event or battle between two livestreamers to attract and engage viewers. The one who gets more viewer engagement, gifts, or votes wins the PK. These friendly competitions bring excitement and large online crowds. Zhengyou (25) says being straight puts him at a disadvantage,

PK game against a gay livestreamer with a gay *dage* is a whole different game. Gay livestreamers know how to be sexy, sassy, and *suo* (搔 flamboyant) to entertain their audience. However, I am not very good at these things. During the competition, after each match, the loser removes a piece of clothing. The first time, I lost badly and had to strip down to my underwear, exposing my skinny figure on camera. Afterward, I could not sleep, worried that my *dag*e would leave. From that moment, I became much more serious about dancing. I learned how to dance competitively to appeal to a gay audience. You do what you must do to succeed.

Almost one-fourth−16 out of 62—of the livestreamers specifically spoke about being complemented and propositioned by gay clients because they were muscular, toned, and physically fit. Therefore, in an ironic contradiction, many of these livestreamers said they must fake authenticity and pretend to be gay in order to obtain large cash tips from clients. They treat those *dage* as cash cows (*tikuanji* 提款機), but emphasized the risks involved. Flirting during the show can get out of hand and affect how the audience perceives the show. They try to focus on positive interactions, being friendly and upbeat, and engage in active listening during the show. This emotional support can strengthen the bond between the livestreamer and their audience, making viewers feel valued and understood. Affective labor can be emotionally taxing. Livestreamers must manage their emotions and maintain their persona, even when tired or not feeling well. They also navigate the fine line between flirtation and inappropriate behavior, ensuring they respect the boundaries of their viewers.

### Livestreaming guilds

All informants admitted they initially had trouble building an audience and often thought of giving up. Some attempts at humor misfired and became major missteps in front of the livestreamed audience. Some hosts were openly accused of selling counterfeit/misleading products or exaggerating the functions of the product. Yinjie (28) left his job as a factory worker in Dongguan in 2017 and moved to Shenzhen to become a livestreamer. He thought it would be easy money. He roared in his first PK competition arranged by his guild to raise his overall number of views to sell lipstick. He solicited votes, but his low-quality microphone buzzed incessantly. At the end of the PK game, it was reported he sold 5,000 lipsticks within 5 min, far more than his opponent, a popular female livestreamer. When the word “champion” leaped onto the screen, Yinjie put his hands together above his head in gratitude and said,

Thank you, “*dage*”! “*Dage*”, please express your satisfaction on the public screen! I sold 5000 lipsticks within five minutes. I am the lipstick KING. I won't be blocked…Please give me more tips, and I will work for you and the guild for affection, sweat, blood, and emotion.”

The two guild directors interviewed admitted they exert control over the performances, setting targets and offering incentives when livestreamers display certain emotions and effectively use humor. For example, livestreamers might be rewarded for their positive and energetic demeanor. In the realm of livestreaming, perpetual triumph remains elusive. Livestreamers exist in a state of perpetual uncertainty, unaware of the precise moment when they might be supplanted or relegated to alternative roles. Their guilds are constrained by adherence to directives issued, obliging them to follow prescribed paths and heed authoritative guidance. Livestreamers must avoid negative or controversial topics that might upset viewers.

Taiyi (31) left his job as a DiDi driver in Guangzhou and set up a Taobao shop in Shenzhen in 2015. He steadily grew sales until it was regularly generating around 300,000 yuan (US$41,600) per month. Sales are cyclical, with peak sales during festival days like Singles Day (November 11), Valentine's Day (February 14), and Lunar New Year (late January-early February). Over a 5-year period, he felt he had finally reached the zenith of his career, but he still had to follow the guild's instructions. He says,

When I started, I trained for a month in the guild's offline room. They taught me about livestreaming and using beauty filters, transforming me from an average-looking guy to a handsome video host. I had to whiten my skin and work out at the gym until my muscles became big. I had to practice being charming and flirting to engage the ladies and men in my audience. I was instructed to be ambiguously gay or straight. Still, they threatened to kick me off if I did not hit their targets. They could mess with my algorithm or stop their friends from pretending to be my fans. It was very stressful, but it made me work harder to connect with my viewers.

Another livestreamer Dandan (23) faced the same risk in livestreaming. He quit his job as a masseur in 2017. He signed on with a guild in Shenzhen and began livestreaming in 2017. He tried selling lipstick and face cream, but the guild said he was not attractive and masculine enough. They demanded he get cosmetic plastic surgery, but did not guarantee he would be successful. Also, Dandan said it was common knowledge that the guild would open fake accounts to make show numbers look better. Although the screen may indicate 100 people are in the chat room, there may only be one or two; the rest are fake. The guild would give him numbers, but it was impossible to know the truth.

At first, the agency (*jingjiren* 经纪人) had me sign a contract and promised that I could become a well-known celebrity. I sold lipstick and face cream, but never went above one thousand followers. One day, the agency suggested I get a nose job to increase my appeal. Since I was broke, the agency gave me a loan but charged me the highest interest rate (*luodai* 裸贷). Unfortunately, the nose job failed… and then I was let go. In hindsight, I was like dim sum (*dianxin* 点心, which literally means food) for those crocodiles.

According to Dandan, the guild treats groups of livestreamers differently. Dandan said female livestreamers must be young, slim, and sexy. Physical attractiveness makes them “algorithmically recognizable” (Gillespie, 2017, p. 2) and thus recommendable to their *dage*. However, straight livestreamers like Dandan claimed the guild limited their algorithmic visibility. If they fell out of favor with the guild, the guild might block them from reaching their *dage* and *dajie* until they submitted to the guild's demands. The guild may demand that they dress and act provocatively or discreetly engage in intimate relationships with followers. Livestreamers typically avoid intimacy with their followers, but exceptions are made—regardless of gender—when they feel desperate for cash. The guild forces the livestreamers to “play the visibility game” (Cotter, 2018) by consciously interacting with their guild's algorithmic infrastructure (Cotter, 2018; Cheney-Lippold, 2011). The guilds extend assistance to livestreamers who monetize big tippers and withhold help from those who don't have such value and utility.

During fieldwork, we observed that one livestreamer, Lufeng (32), consistently misrepresented his geographical origins, claiming to be from Shanghai. After5months of interaction, it was revealed that he was broadcasting from his northern hometown, having never visited metropolises like Beijing, Shanghai, Guangzhou, and Shenzhen. He used software to manipulate his location data, attracting an audience predominantly from Shanghai.

## Discussions

Affective labor offers a conceptual framework for understanding the lifestyles of livestreamers in China. Distinct from emotional labor, affective labor encompasses the feelings and emotions generated through the livestreaming profession, reflecting a deeper engagement with their affect and their jobs as livestreamers. This affective dimension not only shapes the personal lives of livestreamers but also underscores overlooked aspects revealed through conversations with them. In the livestreaming industry, the intersection of gender, class, and power is prominently displayed, influencing both the dynamics within streaming guilds and the self-help initiatives undertaken by streamers. Despite the significant role of guilds in regulating the industry, the Chinese government has yet to establish a comprehensive set of professional ethics for guild governance. Consequently, livestreamers rely heavily on self-help programs, which have proven more effective than reliance on government intervention. These self-help initiatives empower streamers to navigate the complexities of their profession independently, suggesting a pragmatic approach to coping with the demands and challenges of livestreaming. This analysis highlights the necessity of examining affective labor through a multidimensional lens, considering how individual agency, collective guild dynamics, and governmental oversight intersect and influence the evolving landscape of livestreaming in China. Such insights contribute to a deeper understanding of the socio-economic and cultural intricacies that define this burgeoning industry.

### Intersection of gender, class, and power plays out in the livestreaming industry

The empirical findings underscore the strategic utilization of affective labor by male livestreamers residing in Shenzhen, as they navigate their existence with a sense of hope and endeavor to transform their circumstances. For some very popular livestreamers, their annual earnings surge to surpass one million yuan (equivalent to US$154,000). Remarkably, these individuals, lacking formal education, marketable skills, and urban *hukou*, defy conventional expectations by achieving success within the livestreaming domain. This emergent avenue provides an escape route for those who possess street smarts, enabling a seamless transition to e-platforms and the cultivation of digital acumen.

Livestreamers commodify their affective labor to establish themselves as cosmopolitan citizens and nurture their aspiration of urban living (Marshall, 2010). Livestreaming as a profession depends upon sensuous disposition—sights, sounds, mood, and attitude—to significantly enhance a livestreamer's ability to create captivating content that appeals to the sensory experiences of their audience, particularly in a culturally rich city like Shenzhen. Livestreamers must be attuned to all aspects of the visual and auditory elements of their streams, which encompass their vocal tonality, the incorporation of background music or ambient noise, and the overall sound quality of their broadcast. A heightened sensuous disposition enables them to craft a pleasing and immersive auditory landscape for their audience. This interaction predominantly occurs through text or comment communication, evoking a sense of touch or connection between the livestreamer and the audience.

Furthermore, heterosexual male rural migrants feel they are placed into a feminized, subordinate position, becoming objects for the exercise of power by wealthy female or gay patrons, a dynamic negotiated and amplified by guilds. This contributes to the major theoretical contribution to the field of livestreamers and helps us understand how digital platforms are reconfiguring gender politics in contemporary China. Male livestreamers leverage their affective labor to foster intimate connections with their audience, particularly the “big tippers,” and cultivate a close-knit relationship with them. Affective labor is pivotal in fostering this robust bond between livestreamers and their audiences. The livestreamers' consistent management of emotions and interactions with big tippers often cultivates a deep emotional bond between them. When successful, this process leads to more tips, bigger tips, and overall higher income.

The affective labor performed by male bodies serves to re-inscribe gendered hierarchies, as it necessitates the adoption of qualities traditionally aligned with femininity, such as empathy, emotional intelligence, and interpersonal sensitivity. Within sociocultural contexts that valorize traditionally masculine traits—such as assertiveness and independence—men who engage in affective labor often confront challenges to their gender identity. This dynamic not only underscores the persistence of gendered expectations but also reflects the complexities involved in redefining masculinity in modern labor settings.

Affective labor, intrinsically linked to feminine-coded roles, is often undervalued economically, irrespective of the male livestreamers' gender. Men are being feminized, perpetuating a hierarchy where masculine-coded work is elevated in both status and compensation. Even as men increasingly enter domains requiring affective labor, these roles continue to suffer from historical undervaluation, underscoring systemic inequities embedded within labor markets.

Focusing analysis on the intersectionality of affective labor and gender provides a robust theoretical framework for understanding broader societal dynamics. It compels us to question the intrinsic economic and cultural valuations of labor and to recognize how these valuations are shaped by gendered associations. By scrutinizing how affective labor is valued based on its association with femininity, scholars can challenge entrenched economic paradigms and contribute to a reimagining of labor value systems. Such an intervention not only enriches gender theory but also carries the potential to inform policy and practice, advocating for a more equitable recognition and remuneration of affective labor across all gender identities.

The interviewees said they are acutely aware of the power of algorithms and pursue visibility as if playing a game. Algorithms affect social relativities (Bucher, 2017). The rules are established by platform owners and reinforced by algorithms, leading to tactics for engaging and building audiences. Male livestreamers are expected to be physically fit and visually attractive so as to enhance their profitability. Heterosexual male livestreamers encounter challenges in leveraging sexual allure to compete algorithmically with their counterparts who may be more skilled in flirting and being playful. Nevertheless, these men strategically employ their affective labor to sustain their livelihood and foster amicable relations within their guild. They said the bottom line is that it is a challenge to satisfy attendees, but they will do whatever they can to play that part of the game. Cultural nuances significantly influence the livestreaming landscape in China, particularly the underrepresentation of heterosexual male livestreamers.

### Manipulative and passionate guild

The data presented enriches the existing literature on guild involvement with livestreamers in China. Livestreaming guilds often regulate the affective output of livestreamers, prescribing specific emotions at particular times to maximize viewer engagement and revenue generation. In the context of algorithmic control, guilds often collaborate with platforms to ensure a certain level of account activity. Heightened activity levels and viewer engagement can result in the livestreamer being promoted or recommended to a larger audience. The guild's control can be manifested in various ways: dictating the livestreamer's on-screen persona, setting viewer engagement quotas, and mandating strict schedules. The emotional burden of such control can precipitate burnout, stress, and other mental health issues, underscoring the exploitative nature of this industry. Livestreaming guilds in China exercise control over show hosts through training, monitoring, identity manipulation, resource allocation, and algorithm manipulation. This control is designed to maximize viewer engagement and revenue, often at the cost of the livestreamer's autonomy and authenticity.

Almost half of the 62 male livestreamers felt they escaped poverty and found success in urban China with help from the guilds. Livestreaming guilds wield control over entry into this profit-making field (Zhang et al., 2019, p. 8–10). The livestreamers felt the relationship worked because they had a steady job where they could receive bonuses in the form of tips from their wealthiest fans. However, the guilds were also acknowledged to be a source of exploitation. The threat of invisibility may be more common among straight livestreamers who work hard to show loyalty to their agents and guilds. The visibility management (Flyverbom, 2016, p. 112) of the guilds strategically weaponizes information like audience size to render the livestreamers' algorithms invisible to certain *dage* or *dajie*. The asymmetry of power between guilds, platforms, agents, media tycoons, and livestreamers leads to risk-taking activities (Lowe and Tsang, 2019; Tsang, 2023). Many livestreamers say they must take chances lest they become invisible on screen (Beer, 2009). Some interviewees said they were threatened with being blocked and receiving no tips if they did not follow guild instructions. Livestreamers are well aware of this algorithmic power because it prevents transparency and limits visibility in the system (Gillespie, 2017). Bucher (2017) said that algorithmic opacity contributes to an algorithmic imaginary constructed experientially via how users think, talk, and feel about algorithms. However, most of the livestreamers said that if they followed the guild's orders, they were rewarded financially and received favorable audience growth reports. Therefore, most of the livestreamers signed with the guild as their agents to obtain more popularity and exposure on the screen. The tactic for them to succeed is to follow the dictates of the guild.

Most of the livestreamers interviewed do not understand how algorithms work. Factors include audience size, fan “sharing” livestreamer content on other media platforms, fan comments, fan “likes” of livestreamers, numbers regarding “little shells” (*xiaobei* 小貝貝 means digital money), the overall value of the digital gifts received, fans' wealth rank, nobility status, and guardianship. However, the guild manages relationships between agents, commercial companies, and the livestreaming industries (Liu et al., 2021, p. 10). The guild can deliberately obfuscate using algorithms to prevent users from exploiting potential loopholes, like exaggerating the functions of the products. The guild routinely oversees and curates their algorithms, monitoring the performance and general visibility of the livestreamers. Therefore, visibility becomes a reward for the performer and a way to earn more money and increase their popularity, reputation, and legitimacy.

### Self-gain program

From the findings of the article, this article focuses on the affective labor of livestreamers and highlights some exploitative practices within the industry. In the intricate landscape of livestreaming, certain livestreamers operate without formal contractual agreements. These uncharted territories are characterized by ambiguity and opacity, particularly concerning performance expectations set forth by guilds. The elusive benchmarks for success remain shrouded in uncertainty, leaving livestreamers vulnerable to unforeseen consequences. Under what conditions might they face expulsion from the industry, their digital voices silenced by algorithmic intervention? These enigmatic questions persist, casting a veil of unpredictability over the livestreaming domain. The implications of affective labor in digital economies confirm the need for ethical guidelines and industry regulations. The migration patterns of the participants indicate that underprivileged migrants can reinvent themselves as livestreamers and successfully escape poverty. The interviewed migrants employ survival skills and interpersonal communication techniques, colloquially termed “street smarts,” to craft and sustain an appealing online persona that can market products and amass an audience over time. Their migration to urban areas leads them to adopt the lifestyle of their middle-class peers (Cheung and Tsang, 2015; Tsang and Lee, 2013, 2016). These young, unskilled rural migrants, often called “lucky peasants,” shun assistance from government or labor market groups, choosing instead to rely on themselves to achieve their individualistic goals of self-reliance, freedom, and autonomy. Livestreaming success can provide a model for some low-skilled rural migrants to become self-sufficient in the city.

## Conclusion

This study found that the affective labor of livestreamers is anchored in the sensuous disposition or embodied sensations of the urban environments (Crang, 2003). Through parasocial interaction, livestreamer labor also builds relationships with their followers, who believe they have a genuine relationship with them. A broad range of outcomes reflects the complex politics of the livestreamers' desires and exploitations.

This study found that many male migrants working as livestreamers continue to struggle, but not all suffer through long hours, low pay, and feelings of being exploited. In their journey, they have used their own resources, developing street smarts to meet the expectations placed upon them through social dictates (individualized self, freedom, and autonomy). By first migrating to the city and then becoming livestreamers, these heterosexual men have re-situated themselves into the middle of the dominant urban social milieu in ways previously unattainable (Tsang and Lowe, 2019; Tsang, 2021, 2022). The livestreaming industry provides the financial means to consume luxury accouterments that reflect cosmopolitan success, spreading hope and excitement in urban China.

This article contributes to our understanding of this phenomenon in three ways. First, it sheds new light on the experiences of heterosexual male livestreamers in particular and the impact of gender on marketing strategies, exploitation, and algorithmic practices (a set of rules to be followed in calculations in livestreaming) in the guilds. Second, it explores the impact of the guilds on the career trajectories of livestreamers. Finally, it generates new knowledge for the sociological field and helps strengthen the professional ethical standards, intellectual property rights, and labor rights of the livestreamers.

This study contains limitations. A limitation of this study is that it reflects the experiences of a relatively small number of factory workers, taxi drivers, masseurs, salespeople, security guards, and models who became livestreamers. Their experiences may be reflective of only a specific region of Shenzhen and other major metropolitan areas in China. Therefore, this data cannot be generalized across the entire country, as it is primarily applicable to urban settings. It may not be applicable to major rural areas. Future research should be more inclusive and more representative of these emerging industries in China.

## Data Availability

The raw data supporting the conclusions of this article will be made available by the authors, without undue reservation.
